# Risk of work-related health problems among community health agents

**DOI:** 10.47626/1679-4435-2025-1490

**Published:** 2025-11-07

**Authors:** Luiz Henrique Rodrigues de Souza, Ruth Emanuele Silva Andrade, Lucineia de Pinho, Narciso Ferreira dos Santos Neto

**Affiliations:** 1 Postgraduate Program in Computational Modeling and Systems, Universidade Estadual de Montes Claros (Unimontes), Montes Claros, MG, Brazil.; 2 School of Medicine, Unimontes, Montes Claros, MG, Brazil.; 3 Postgraduate Program in Primary Health Care, Unimontes, Montes Claros, MG, Brazil.

**Keywords:** occupational health, lifestyle, community health workers, saúde ocupacional, estilo de vida, agentes comunitários de saúde

## Abstract

**Introduction:**

Work-related health problems may include physical injuries, such as musculoskeletal disorders, as well as psychosocial issues, including isolation, stress, and anxiety. Assessing the risk of work-related health problems among community health agents is essential for identifying contributing factors and providing evidence to support the development of public policies and intervention strategies.

**Objectives:**

To evaluate the risk of work-related health problems among community health agents and their associated factors.

**Methods:**

A cross-sectional, descriptive, and analytical study was conducted in 2018 with 675 community health agents from the city of Montes Claros, Minas Gerais, Brazil. Participants completed a self-administered questionnaire addressing sociodemographic, occupational, and lifestyle characteristics. Work-related problems were assessed using the Work-Related Health Problems Assessment Scale. Data were analyzed using absolute and relative frequencies and Pearson’s chi-square test.

**Results:**

Most participants were women (83.7%), under 40 years old (65.2%), and had worked as a community health agent for less than 5 years (56.6%). In terms of lifestyle, 73.9% were moderately active, 70.1% reported having a healthy diet, and 35.6% experienced stress. High levels of work-related physical (86.6%), psychological (50.5%), and social (50.1%) problems were observed. A higher prevalence of health problems was associated with female sex, longer service time (> 5 years), physical inactivity, poor sleep quality, and inadequate stress management.

**Conclusions:**

A high prevalence of work-related health problems was identified among community health agents, associated with sociodemographic, occupational, and lifestyle factors.

## Abstract (pt)

**Introdução:**

Os danos à saúde relacionados ao trabalho podem incluir lesões físicas, como distúrbios musculoesqueléticos, e problemas psicológicos e sociais, como isolamento, estresse e ansiedade. Analisar o risco de danos à saúde entre agentes comunitários de saúde é fundamental para identificar os fatores contribuintes, fornecendo subsídios essenciais para a formulação de políticas públicas e estratégias de intervenção.

**Objetivos:**

Analisar os riscos de danos à saúde relacionados ao trabalho entre agentes comunitários de saúde e seus fatores associados.

**Métodos:**

Estudo transversal, descritivo e analítico, realizado com 675 agentes comunitários de saúde de Montes Claros, Minas Gerais, em 2018. Foi utilizado um questionário autoaplicado com questões sociodemográficas, laborais e de estilo de vida. Os danos relacionados ao trabalho foram avaliados por meio da Escala de Avaliação dos Danos Relacionados ao Trabalho. Os dados foram analisados utilizando frequências absolutas e relativas e o teste do qui-quadrado de Pearson.

**Resultados:**

A maioria dos participantes era mulher (83,7%), tinha menos de 40 anos (65,2%) e trabalhava na função há menos de 5 anos (56,6%). Em relação ao estilo de vida, 73,9% eram moderadamente ativos, 70,1% tinham alimentação saudável e 35,6% relataram estresse. Foram observados altos níveis de danos físicos (86,6%), psicológicos (50,5%) e sociais (50,1%). A maior prevalência de danos esteve associada às variáveis sexo feminino, mais de 5 anos de serviço, ausência de atividade física, problemas de sono e falta de manejo do estresse.

**Conclusões:**

Houve alta prevalência de danos relacionados ao trabalho, associada a fatores sociodemográficos, laborais e de estilo de vida.

## INTRODUCTION

Work-related health problems encompass physical injuries, such as musculoskeletal disorders, as well as psychological and social issues, including isolation, stress, and anxiety. This topic has gained increasing attention in the field of occupational health, as such conditions adversely affect not only workers’ health status and quality of life but also their safety and productivity at work.^^1^,^2^^ During professional practice, health problems may arise from intrinsic aspects of the work process, such as inadequate ergonomic conditions and exposure to toxic substances, which can lead to musculoskeletal pain, arthritis, tendinitis, silicosis, and temporary or permanent hearing loss. In addition, pressure to be productive is often cited as a contributing factor to mental health disorders among workers.^^3^^

Extrinsic factors, in turn, relate to workers’ lifestyle habits. Healthy habits – such as a balanced diet, regular physical activity, adequate sleep, low consumption of harmful substances (especially tobacco and alcohol), and adequate stress management – not only improve physical and mental health but also enhance productivity and efficiency in the workplace.^^4^-^6^^

A healthy lifestyle promotes overall well-being and quality of life both inside and outside the workplace. Workers who adopt healthier habits often experience substantial improvements in physical health, with reduced risks of noncommunicable chronic diseases associated with harmful behaviors, as well as marked reductions in depression, anxiety, fatigue, and work-related conditions such as burnout syndrome and post-traumatic stress disorder. These factors contribute to greater job satisfaction and maintaining a healthy work-life balance.^^5^,^7^^

Analyzing the risk of work-related health problems among community health agents (CHAs) is essential to identify contributing factors and provide evidence for the development of effective public policies and intervention strategies. Therefore, the aim of this study was to analyze the risk of work-related health problems among CHAs and their associated factors.

## METHODS

This cross-sectional, descriptive, and analytical study was part of the research project “Working Conditions and Health of Community Health Agents in Northern Minas Gerais,” conducted by the State University of Montes Claros (Unimontes).

The target population comprised the 797 CHAs working in the 135 Family Health Strategy (FHS) teams in Montes Claros, Brazil, in 2018. All CHAs were invited to participate in the study. The inclusion criterion was CHAs who were actively performing their duties; those who were on leave or pregnant at the time of data collection were excluded.

Data were collected by health professionals and undergraduate research assistants at the Regional Reference Center for Workers’ Health (*Centro de Referência Regional em Saúde do Trabalhador*) in Montes Claros, on weekday mornings, between August and October 2018. Participants completed a structured, self-administered questionnaire covering sociodemographic, occupational, and lifestyle characteristics. The following variables were analyzed: sociodemographic data – sex, age, years of education, marital status, skin color, income, and religiosity; occupational characteristics – length of service as a CHA, employment relationship and lifestyle – physical activity, diet, overweight, smoking, alcohol consumption, sleep quality, and stress management.

The presence of work-related health problems was assessed using the Work-Related Health Problems Assessment Scale (*Escala de Avaliação dos Danos Relacionados ao Trabalho*, EADRT), which is one of the scales that make up the Work and Illness Risk Inventory (*Inventário do Trabalho e Riscos de Adoecimento*) – a self-administered tool created and validated in Brazil which assesses the relationship between work and the risk of illness across different dimensions. The EADRT includes 29 items distributed across three domains: physical problems (items 1–12), psychological problems (items 13–22), and social problems (items 23–29). The items are rated on a 7-point scale referring to the frequency of symptoms over the past 6 months: 0 = never, 1 = once, 2 = twice, 3 = three times, 4 = four times, 5 = five times, and 6 = six or more times. Scores above 4.1 indicate the presence of occupational disease; 3.1–4.0 indicate severe risk; 2.0–3.0 indicate critical risk; and below 1.9 indicate tolerable risk.^^8^^

For analytical purposes, the variables were categorized as follows: sociodemographic characteristics – sex (male, female), age (≤ 40, > 40 years), education (≤ 11, > 11 years), marital status (has a partner, does not have a partner), skin color (White, non-White), income (< 2, 2–3, > 3 minimum wages), and religiosity (yes, no); occupational characteristics – time working as a CHA (≤ 5, > 5 years) and employment relationship (civil servant/duly appointed, contractor/hired under the Brazilian Consolidation of Labor Laws (CLT) regime/service provider); and lifestyle characteristics – physical activity (no, yes), diet (healthy, unhealthy), overweight (no, yes), smoking (no, yes), alcohol consumption (no, yes), sleep quality (poor, good), and stress management (no, yes). The outcome variable was the total EADRT score, dichotomized as “up to the mean” and “above the mean” for the study population.

Descriptive analyses were performed using absolute and relative frequencies. Bivariate analyses between the dependent variable and each independent variable were conducted using Pearson’s chi-square test. Crude and adjusted prevalence ratios (PRs) and 95%CIs were estimated. Variables with p ≤ 0.20 were included in the multiple analysis. Multiple regression was performed using a Poisson model with robust variance to estimate the magnitude of associations through crude and adjusted PRs, adopting a 5% significance level. Model fit quality was assessed using the deviance test. All analyses were performed using the *Statistical Package for the Social Sciences* (SPSS), version 20.0.

The project “Working Conditions and Health of Community Health Agents in Northern Minas Gerais” was approved by the Research Ethics Committee of Unimontes (approval no. 56). All participants were informed in advance about the study objectives and the preservation of anonymity and provided written informed consent prior to participation.

## RESULTS

Of the 797 CHAs in Montes Claros, 122 (15.3%) were excluded from the study because they were not actively performing their duties, were pregnant, had been working as a CHA for less than 1 year, were on maternity leave, or were temporarily away from work. Thus, a total of 675 CHAs were interviewed.

Among the participants, there was a predominance of women (83.7%), individuals younger than 40 years (65.2%), and those with fewer than 11 years of education (56.7%). Regarding occupational characteristics, most had worked as a CHA for less than 5 years (56.6%) and were hired as a contractor or under the CLT regime (74.1%).

In terms of lifestyle, 73.9% of the CHAs reported being moderately active, and 70.1% reported maintaining a healthy diet. Additionally, 60.4% were overweight, 37.7% consumed alcohol, and 35.6% reported experiencing stress (Table 1).

**Table 1:** Characteristics of the sample of community health agents, Northern Minas Gerais (n = 675), 2018

Variables	n	%
Sex		
Male	110	16.3
Female	565	83.7
Age, years		
≤ 40	440	65.2
> 40	235	34.8
Education, years		
≤11	383	56.7
> 11	292	43.3
Marital status		
Has a partner	403	59.7
Does not have a partner	272	40.3
Skin color		
White	87	12.9
Non-White	588	87.1
Income, minimum wages		
< 2	353	52.3
2-3	185	27.4
> 3	137	20.3
Time working as a CHA, years		
≤ 5	382	56.6
> 5	293	43.4
Religious		
Yes	569	84.4
No	105	15.6
Employment relationship		
Civil servant/duly appointed	175	25.9
Contractor/CLT/service provider	500	74.1
Physical activity, moderately active		
Yes	498	73.9
No	176	26.1
Diet		
Healthy	432	70.1
Not healthy	184	29.9
BMI, overweight		
No	267	39.6
Yes	407	60.4
Smoking		
No	637	94.5
Yes	37	5.5
Alcohol consumption		
No	420	62.3
Yes	254	37.7
Sleep quality		
Good	400	59.3
Poor	274	40.7
Stress management		
Yes	434	64.4
No	240	35.6

CLT = hired under the Brazilian Consolidation of Labor Laws regime.

Chart 1 presents the descriptive analysis of the EADRT items. The EADRT is composed of three domains: physical problems (M = 21.24; SD = 15.28), social problems (M = 8.57; SD = 10.65), and psychological problems (M = 9.87; SD = 12.47). In the physical problems domain, the items with the highest scores – classified as critical – were “body pain,” “headache,” “back pain,” “sleep disturbances,” and “leg pain.”

**Chart 1:** Work-Related Health Problems Assessment Scale

Item	Mean	SD	Item classification
Physical problems	21.24	15.28	–
Q1 Body pain	2.74	2.10	Critical
Q2 Arm pain	1.86	2.00	Tolerable
Q3 Headache	2.89	2.20	Critical
Q4 Respiratory disorders	0.91	1.50	Tolerable
Q5 Digestive disorders	0.97	1.50	Tolerable
Q6 Back pain	2.69	2.30	Critical
Q7 Hearing disorders	0.55	1.30	Tolerable
Q8 Changes in appetite	1.34	1.90	Tolerable
Q9 Eye disorders	1.25	1.80	Tolerable
Q10 Sleep disturbances	2.09	2.10	Critical
Q11 Leg pain	2.89	2.20	Critical
Q12 Circulatory disorders	1.06	1.70	Tolerable
Social problems	8.57	10.65	–
Q13 Insensitivity toward colleagues	1.00	1.50	Tolerable
Q14 Difficulty maintaining relationships outside work	0.86	1.50	Tolerable
Q15 Desire to be alone	1.60	1.90	Tolerable
Q16 Family conflicts	1.05	1.50	Tolerable
Q17 Aggressiveness toward others	0.66	1.30	Tolerable
Q18 Difficulty maintaining friendships	0.61	1.20	Tolerable
Q19 Impatience with people in general	1.25	1.70	Tolerable
Psychological problems	9.87	12.47	–
Q20 Bitterness	0.75	1.40	Tolerable
Q21 Feeling of emptiness	1.08	1.70	Tolerable
Q22 Feeling of helplessness	1.04	1.70	Tolerable
Q23 Bad mood	1.34	1.70	Tolerable
Q24 Desire to give up everything	1.31	1.90	Tolerable
Q25 Sadness	1.42	1.90	Tolerable
Q26 Irritability	1.27	1.80	Tolerable
Q27 Feeling of abandonment	0.94	1.70	Tolerable
Q28 Self-doubt about ability to perform tasks	1.33	1.70	Tolerable
Q29 Loneliness	0.96	1.70	Tolerable
Overall score	39.68	34.60	–

EADRT scores were classified into four categories: above 4.0 = negative evaluation, indicating the presence of occupational disease; 4.0–3.1 = moderate to frequent evaluation, considered severe; 3.0–2.0 = moderate evaluation, considered critical; and below 1.99 = positive evaluation, considered tolerable.

Among CHAs, the prevalence of occupational disease was 86.6% for the physical problems domain, 50.5% for psychological problems, and 50.1% for social problems (Table 2).

**Table 2:** Risk of physical, psychological, and social problems according to the EADRT among community health agents, Northern Minas Gerais (n = 675), 2018

Variables	n	%
Physical		
Tolerable	46	6.8
Critical	32	4.7
Severe	12	1.8
Presence of occupational disease	582	86.6
Psychological		
Tolerable	214	31.8
Critical	89	13.2
Severe	30	4.5
Presence of occupational disease	340	50.5
Social		
Tolerable	221	22.8
Critical	72	10.7
Severe	43	6.4
Presence of occupational disease	337	50.1
EADRT		
Up to the mean	416	61.9
Above the mean	256	38.1

EADRT = Work-Related Health Problems Assessment Scale.

**Figure 1:**
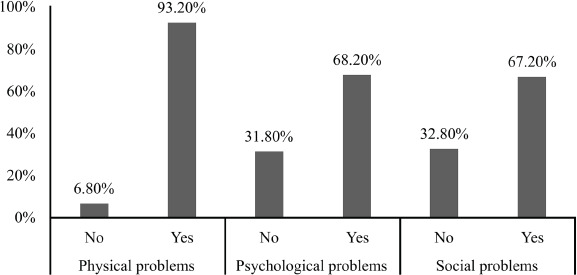
Prevalence of health problems according to the Work-Related Health Problems Assessment Scale.

In the dichotomized analysis of work-related health problems among CHAs, a lower level of impairment was observed in the social problems domain (32.8%) (Figure 1).

Table 3 presents the estimated magnitudes (crude and adjusted PRs) of the associations between the EADRT and sociodemographic, occupational, and lifestyle factors. A positive and statistically significant association was found between the EADRT and the variables sex, time working as a CHA, physical activity, sleep quality, and stress management.

**Table 3:** Prevalence and crude and adjusted PR for the Work-Related Health Problems Assessment Scale, according to sociodemographic, occupational, and lifestyle variables of CHAs (n = 675), Northern Minas Gerais, 2018

Variable	Prevalence (above the mean)	Crude PR		Adjusted PR	
	n (%)	(IC95%)	p-value	(IC95%)	p-value
Sex			0,001		0,012
Male	26 (24.1)	1		1	
Female	230 (40.8)	1.14 (1.06–1.22)		1.09 (1.02–1.16)	
Age, years			0.937		–
≤ 40	166 (38.0)	1		–	
> 40	90 (38.3)	1.00 (0.95–1.06)		–	
Education, years			0.933		–
≤ 11	145 (38.0)	1		–	
> 11	111 (38.3)	1.00 (0.95–1.06)		–	
Marital status			0.267		–
Has a partner	160 (39.8)	1		–	
Does not have a partner	96 (35.6)	0.97 (0.92–1.02)		–	
Skin color			0.660		–
White	35 (40.2)	1		–	
Non-White	221 (37.8)	0.98 (0.91–1.06)		–	
Income, minimum wages			0.464		–
< 2	133 (38.0)	1		–	
2–3	76 (41.1)	1.02 (0.96–1.09)		–	
> 3	47 (34.3)	0.97 (0.91–1.04)		–	
Time working as a CHA, years			0.000		0.000
≤ 5	103 (27.2)	1		1	
> 5	153 (52.2)	1.20 (1.14–1.26)		1.15 (1.09–1.20)	
Religious			0.325		–
Yes	211 (37.2)	1		–	
No	44 (42.3)	1.04 (0.96–1.12)		–	
Employment relationship			0.000		–
Civil servant/duly appointed	93 (53.1)	1		–	
Contractor/CLT/service provider	163 (32.8)	0.87 (0.82–0.92)		–	
Physical activity			0.007		0.019
Yes	174 (35.1)	1		1	
No	82 (46.6)	1.09 (1.02–1.15)		1.07 (1.01–1.13)	
Diet			0.322		–
Health	159 (37.1)	1		–	
Not healthy	76 (41.3)	1.03 (0.97–1.09)		–	
BMI, overweight			0.210		–
No	93 (35.1)	1		–	
Yes	162 (39.9)	1.04 (0.98–1.09)		–	
Smoking			0.703		–
No	243 (38.3)	1		–	
Yes	13 (35.1)	0.98 (0.87–1.10)		–	
Alcohol consumption			0.773		–
No	161 (38.5)	1		–	
Yes	95 (37.4)	0.99 (0.94–1.05)		–	
Sleep quality			0.000		0.000
Good	96 (24.1)	1		1	
Poor	160 (58.6)	1.28 (1.22–1.34)		1.21 (1.15–1.27)	
Stress management			0.000		0.000
Yes	122 (28.2)	1		1	
No	134 (56.1)	1.22 (1.16–1.28)		1.11 (1.05–1.17)	

*Deviance*: 87,786; p = 0,132. CHA = community health agent; CLT = hired under the Brazilian Consolidation of Labor Laws regime; PR = prevalence ratio.

In the adjusted multiple model, higher EADRT prevalence – compared with participants whose total scores were below the mean – was observed among women (PR = 1.09; 95%CI: 1.02-1.16), those working as a CHA for more than 5 years (PR = 1.15; 95%CI: 1.09-1.20), those who did not engage in physical activity (PR = 1.07; 95%CI: 1.01-1.13), those who reported poor sleep quality (PR = 1.21; 95%CI: 1.01-1.13), and those who did not manage stress effectively (PR = 1.11; 95%CI: 1.05-1.17).

## DISCUSSION

The results of this study revealed a high prevalence of physical, social, and psychological problems among CHAs. In the domain-specific analysis, physical problems were classified as an occupational disease in 86.6% of participants. In the item-by-item analysis, all items from this domain showed a mean classification of “tolerable,” except for “body pain,” “headache,” “back pain,” “leg pain,” and “sleep disturbances,” which were rated as critical.

In a previous study conducted with CHAs, physical problems were classified as “critical” – less negative than the classification found in the present study. However, structured interviews from that study revealed that participants tended to normalize pain and physical strain (such as walking long distances and being exposed to adverse weather conditions) as inherent aspects of their work activities.^^9^^

In a study involving FHS workers in the state of Mato Grosso do Sul, the risk classification for physical problems was “severe,” with “back pain,” “body pain,” and “sleep disturbances” identified as the items with the poorest evaluations (indicative of occupational disease).^^10^^ In contrast, a study conducted with primary health care (PHC) professionals in the city of Patos de Minas, Minas Gerais, reported an overall satisfactory evaluation for physical problems; however, specific items – body pain (M = 2.8), headache (M = 2.3), back pain (M = 2.4), and leg pain (M = 2.5) – were classified as critical, consistent with the findings of the present study.^^11^^

The physical problems observed among CHAs are partly due to their continuous exposure to physically and emotionally demanding situations, as their activities are conducted predominantly outside health units. Exposure to poverty and violence, the long distances traveled daily for home visits, and the challenging geographic characteristics of the territories they serve contribute to cumulative strain over time.^^9^^ Excessive use of the musculoskeletal system, combined with insufficient recovery time, may account for the higher frequency of pain-related symptoms compared with other physical conditions.^^11^^ Musculoskeletal disorders have been identified as a major cause of sick leave among PHC workers, highlighting the potential negative impact of physical problems in the workplace, as well as the individual limitations and repercussions that may result from such conditions.^^10^^

Psychological problems were classified as an occupational disease in 50.5% of the sample. In the item-level analysis, all components of this domain showed a mean classification of “tolerable.” A previous study conducted with health professionals also identified a negative classification for psychological problems (critical), as well as negative ratings for specific items, including “sadness” (4.08 – occupational disease), “desire to give up everything” (3.44 – severe), and “feeling of abandonment” (3.41 – severe).^^10^^

Conversely, some studies in the literature have reported satisfactory evaluations for psychological problems.^^12^^ In a study conducted with CHAs working in FHS teams in Rio Grande do Sul, psychological problems were classified as “tolerable.” However, semi-structured interviews revealed reports of “diseases of the soul” and depressive symptoms.^^9^^ In another study, “sadness” was also classified as critical, together with “bad mood,” which was classified as severe.^^11^^

During their work activities, CHAs are exposed to several situations that generate emotional distress, as they deal directly with the needs and vulnerabilities of community members, including death, illness, violence, and poverty. These professionals are often, although unfairly, held responsible for providing support and resolving such situations. Continuous exposure to emotional distress frequently leads these experiences to be perceived as inherent to the profession, resulting in the neglect of CHA’s emotional suffering under the assumption that they must always be fully capable of handling every aspect of their work.^^9^^ The lack of coping strategies to address the sources of psychological distress has individual consequences for these workers, leading to the development of mental disorders such as depression, anxiety, and burnout syndrome, while also compromising productivity and work performance.^^13^^

Social problems were classified as an occupational disease in 50.1% of the sample, and all items related to this domain were rated as “tolerable” based on the overall mean score. Another study also identified a negative classification for social problems (severe), as well as for specific items such as “desire to be alone” (3.49 – severe), “impatience with people in general” (3.31 – severe), and “difficulty maintaining relationships outside work” (2.90 – severe).^^10^^ Similarly, a study conducted with PHC workers found that “impatience with people in general” (M = 2.7) was negatively evaluated and classified as critical.^^11^^

Conversely, in some studies, social problems have been classified as “tolerable” among most participants.^^9^,^14^^ Nevertheless, such conditions should be given due importance in the prevention of work-related health issues, as they may compromise CHAs’ ability to build and maintain high-quality social and family relationships, thereby increasing their susceptibility to depressive symptoms and other emotional disorders.^^10^^

Overall EARDT scores above the mean were associated with female sex, longer time working as a CHA, physical inactivity, poor sleep quality, and inadequate stress management, indicating these characteristics are risk factors for the development of work-related health problems.

A study conducted with hospital workers also identified significantly higher mean scores for work-related psychological problems among women (1.59) compared to men (0.80).^^15^^ Women experience physical, social, and psychological disparities compared to men, making them more vulnerable to occupational hazards. Physiologically, women have distinct neuromuscular, metabolic, and morphological characteristics, which generally result in lower physical fitness than men. Reduced muscular strength and endurance make women more susceptible to fatigue and, consequently, to physical damage.^^16^^ While men are at higher injury risk in occupations in the industry sector involving physical exposure, women are at higher injury risk in health care occupations due to the intense physical demands of such work, including lifting patients and performing repetitive tasks.^^17^^

Socially, women are subjected to greater social pressure and stress due to the need to fulfill multiple roles, balancing professional demands with personal and family responsibilities.^^18^^ In addition, women tend to receive less organizational support, which negatively affects workplace well-being and increases the risk of illness, as demonstrated in a study conducted with Brazilian workers.^^19^^ In a study involving health care professionals in Bologna, women showed a higher prevalence of diagnosed mental disorders, particularly depressive disorders (p < 0.001).^^13^^

A study analyzing reported cases of work-related mental disorders in the state of São Paulo found a higher number of notifications among women.^^20^^ Furthermore, a study conducted with CHAs in the city of Fortaleza found that female professionals experienced higher rates of domestic violence and community violence within the neighborhoods where they live or work, thereby increasing their risk of developing common mental disorders.^^21^^ The combination of these factors increases the risk of burnout and other work-related complications among women, underscoring the need for occupational health policies that are more sensitive to gender issues.

In the present study, a significant association was observed between longer length of service and a risk of work-related health problems above the mean. Similarly, a study involving surgical nurses identified a correlation between longer work experience and a higher risk of physical problems (p = 0.082).^^22^^ An analysis on the impact of organizational support found that workers with up to 2 years of service perceived greater organizational support than those with more than 3 years in the position, and this factor was associated with a lower risk of work-related physical, social, and psychological problems.^^23^^ Although longer service time may improve CHAs’ performance in certain functions and strengthen their bond with the community – thereby facilitating their work –, it also increases their exposure time to occupational hazards, resulting in greater physical and psychological strain.^^24^,^25^^ Therefore, longitudinal health measures aimed at preventing the development of long-term physical and emotional disorders could be beneficial for this population.

In this study, physical inactivity was associated with a higher risk of work-related health problems. Similarly, a study conducted with hospital nurses found that physical inactivity was related to social problems, particularly higher levels of social isolation and difficulty maintaining interpersonal relationships.^^14^^ In Taiwan, lack of exercise was significantly associated with the development of burnout syndrome and exhaustion among health care professionals.^^5^^ Physical inactivity represents a self-care deficit behavior that not only increases the risk of illness among workers but also negatively impacts their professional performance.^^14^^ Conversely, engaging in daily moderate physical activity has been associated with reductions in symptoms of tension (p < 0.01), anger (p = 0.02), fatigue (p < 0.01), depression (p < 0.01), and mental confusion (p < 0.01), demonstrating the positive effects of exercise on workers’ mood and well-being.^^26^^

In this study, poor sleep quality was also associated with high risk of work-related health problems. A previous study also found that poor sleep quality was associated with an increased risk of physical (p = 0.019) and social problems (p = 0.000).^^22^^ When sleep is insufficient in either quality or quantity, it leads to reduced performance and productivity, decreased concentration and attention, and elevated physiological stress, compromising both the worker’s physical integrity and the safety of others. Moreover, inadequate sleep affects the body’s metabolism and has been linked to chronic diseases such as obesity and diabetes, as well as musculoskeletal disorders such as low back pain.^^27^,^28^^ In Taiwan, unsatisfactory sleep was significantly associated with higher rates of depression, exhaustion, and burnout syndrome among health care professionals.^^5^^ A study evaluating the quality of life of nurses found that sleep disorders increased the likelihood of poor quality of life by 3.15 times.^^14^^ The combination of these factors may contribute to a greater predisposition to work-related health problems, as professionals who do not sleep well tend to experience higher levels of physical and emotional exhaustion.

Lack of stress management was also associated with a risk of work-related health problems above the mean. A study conducted with women in higher education management found that those with higher levels of overload and stress were more likely to exhibit more harmful physical and emotional symptoms related to work. Conversely, participants who adopted stress management strategies reported lower stress levels; notably, these strategies tended to be individual in nature, including psychotherapy, integrative therapies, physical activity, and taking breaks during work.^^18^^ Promoting healthy stress management practices among CHAs is crucial, as prolonged exposure to stressful conditions may lead them to adopt maladaptive coping strategies. This behavior was illustrated in a study involving health professionals in Paris, which identified a high prevalence of tobacco, tranquilizer, and opioid use during the COVID-19 pandemic – a particularly stressful period.^^29^^

The present study revealed that CHAs in Montes Claros experience high levels of physical, psychological, and social problems related to work. These findings underscore the urgent need for interventions aimed at promoting the health and well-being of these workers. Implementing programs that encourage regular physical activity, improve working conditions, foster effective stress management strategies, and promote adequate sleep quality may significantly reduce the risk of occupational diseases. In addition, developing and implementing occupational health policies that promote healthier and more sustainable work environments is essential. Such policies should address not only physical health but also the psychological and social dimensions of well-being. A comprehensive approach to workers’ health is crucial to ensure both the effectiveness and sustainability of the services provided by CHAs, directly contributing to improving the quality of health care delivered to the community.

## CONCLUSION

The risk of work-related health problems among the CHAs evaluated in this study was high. The factors associated with the higher incidence of these risks involved sociodemographic, occupational, and lifestyle characteristics. These findings highlight the need for integrated public policies and multidisciplinary actions focused on preventing work-related health problems and promoting a healthier, more resilient workforce.
